# Evaluation of the reliability and validity of a Caregivers’ Complementary Feeding Practice Scale (CCFPS) for children aged 6–23 months in urban areas of China

**DOI:** 10.7189/jogh.15.04170

**Published:** 2025-06-20

**Authors:** Qiong Wu, Na Meng, Lihui Xie, Lin Li, Yiwen Huang, Yanfeng Zhang

**Affiliations:** 1 Department of Integrated Early Childhood Development, Beijing Municipal Key Laboratory of Child Development and Nutriomics, Capital Institute of Pediatrics, Beijing, China; 2 Department of Health Quarantine, Beijing Customs District P.R.China, Beijing, China; 3 Beijing KidsHome Children Development Centre, Beijing, China; 4 Child Healthcare Center, Capital Center for Children’s Health, Capital Medical University, Beijing, China

## Abstract

**Background:**

Caregivers’ feeding practices significantly influence children’s eating behaviours. We previously developed a Caregivers’ Complementary Feeding Practice Scale (CCFPS) for children aged 6–23 months in China. We aimed to comprehensively assess the CCFPS's reliability and validity, thus establishing a reliable and robust tool for evaluating caregivers’ feeding practices.

**Methods:**

Through convenience sampling, we recruited 928 caregivers from community health centres. We assessed the scale’s reliability with Cronbach's alpha and split-half analysis. Additionally, we used the content validity index (CVI) for content validity. Lastly, we examined structural validity with exploratory factor analysis (EFA) and confirmatory factor analysis (CFA).

**Results:**

The scale's Cronbach’s alpha coefficient was 0.783, and its split-half reliability coefficient reached 0.750. The CVI was 0.910. EFA supported the scale's six-factor structure, which explained 51.43% of the total variance. The CFA results demonstrated that the fit indices of the model were higher than the acceptable level, indicating the scale provides a good level of validity.

**Conclusions:**

The CCFPS is a reliable and valid tool for assessing caregivers’ feeding practices with infants and young children aged 6 to 23 months in urban China, suitable for research and clinical applications.

The first two years of life are crucial for children’s physical growth and development, laying the foundation for their future health [1]. Poor eating behaviours in this period can also increase the risk of malnutrition in children, stunting, and obesity in adulthood [2]. According to reports, the global prevalence of children's unhealthy eating habits, including selective eating, food neophobia, and anorexia nervosa, ranges from 5.6% to 59.0% [3–5], while in China it varies between 25.0% and 64.7% [6–8].

The complementary feeding period, generally for 6–23 months of age, is crucial for establishing healthy eating habits [9], with parents playing a significant role in children's eating behaviours development [10]. Particular attention should be given to caregivers’ feeding behaviours for children during this period. Caregivers’ feeding behaviour refers to the specific strategies or techniques used while feeding their children [11]. Children's eating behaviour is primarily influenced by caregivers’ feeding practices [12]. Previous studies have demonstrated a positive correlation between picky eating behaviour in children and coercive feeding practices by parents [13], as well as a negative correlation with parents' promotion of balanced diet feeding practices [14]. In addition, caregivers’ feeding practices significantly contribute to the development of childhood obesity and being overweight [15], exerting a direct influence on the occurrence or prevention of excessive weight gain among children [16]. In recent years, childhood obesity has become one of the most significant public health challenges [17,18]. Therefore, identifying and improving parents' feeding practices is key to preventing childhood obesity.

Various assessment tools have been extensively studied to evaluate caregivers’ feeding behaviours. However, most of the tools assess caregivers of children aged ≥2-year, such as the Child Feeding Questionnaire [19], the Caregiver’s Feeding Style Questionnaire [20], the Parental Feeding Style Questionnaire [21], the Comprehensive Feeding Practices Questionnaire [22], and the Preschool Feeding Questionnaire [23]. For children aged <2 years, the existing Infant Feeding Questionnaire [23], within the Parent Feeding Behavior Scale, demonstrates low reliability, while the Infant Feeding Style Questionnaire [24] lacks universality as it primarily targets low-income African Americans. Shanghai Jiao Tong University developed the Young Child Feeding Questionnaire in China, which evaluated caregivers’ attitudes and feeding practices towards children aged 6–18 months. However, further improvements are required regarding internal consistency and validity testing [25]. Therefore, there is a lack of a universally accepted measurement scale for evaluating the complementary feeding practices of Chinese caregivers with children aged 6–23 months. To fill this gap, we developed a Caregivers’ Complementary Feeding Practice Scale (CCFPS) for caregivers with children aged 6–23 months following the standard scale development procedure in 2016 [26]. We report the scale’s reliability and validity to establish a robust instrument for assessing the feeding practices among Chinese caregivers.

## METHODS

### Study design

We carried out this cross-sectional survey between November 2016 and January 2017 in three cities in China (Harbin (Northeast), Xiamen (Southeast) and Xi’an (Northwest)). Participants were primary caregivers of children aged 6–23 months who came for regular health check-ups or vaccinations at community health service centres and were recruited using a convenience sampling method. We excluded caregivers with children who had neural tube defects, metabolic disease or congenital heart disease, or those who were unwilling to participate.

### Sample size

The sample size calculation was based on a study conducted by Tinsley, which stated that the sample size of a scale should be approximately 5–10 times the number of items [27]. Given that our scale consists of 31 items, and considering regional differences, we aimed to recruit 300 participants in each region.

### Survey instrument

The survey instrument in this study included two sections. The first section collected data on demographic characteristics (*i.e.* child’s gender, date of birth and place, child-caregiver relationship, caregivers’ education and occupation, household structure, and monthly income), and the second section was the CCFPS scale.

### CCFPS

Our research team developed the evaluated CCFPS scale in 2016, and the standard scale development process of the CCFPS scale was reported in our previous paper [26]. We first developed eight dimensions by reviewing the literature on caregivers’ feeding practices and discussing with six experts. Based on the dimensions and referring to existing scales of caregivers’ feeding practices (*i.e.* the Infant Feeding Style Questionnaire, Infant Feeding Questionnaire, Child Feeding Questionnaire, Comprehensive Feeding Practices Questionnaire, Preschool Feeding Questionnaire, Parental Feeding Style Questionnaire, and Young Child Feeding Questionnaire), we developed an item pool. The item pool consists of 73 items, adapted from some existing scales and self-developed based on Chinese context. Then, we conducted two rounds of expert consultations via email using the Delphi method. Based on the consultation results, we excluded 31 items that lacked universality or had unclear and inaccurate expressions. Meanwhile, we invited 20 caregivers to assess the items and revised those that were hard to comprehend, semantically unclear, or likely to cause misinterpretation. Subsequently, we carried out a preliminary evaluation of the scale with 386 caregivers whose children aged 6–23 months in Beijing. At this stage, exploratory factor analysis (EFA) combined with parallel analysis indicated that it was more appropriate to extract six factors. Moreover, 12 items with a correlation coefficient less than 0.3 (*P* < 0.05), and three items with factor loadings >0.3 were excluded. Finally, the CCFPS scale with six dimensions and 27 items was developed. The six dimensions comprised pressure to eat, concern about child undereating, emotional feeding or instrumental feeding, prompting and encouragement to eat, restriction, and concern about child overeating [26]. Each dimension consisted of 3–6 items. The 5-Point Likert-type scale was used for each item (1 = Never, 2 = Rarely, 3 = Sometimes, 4 = Often, and 5 = Always), with higher mean scores indicating a more pronounced tendency of caregivers to adopt this feeding approach for their children [26].

### Data collection

From November 2016 to January 2017, we used a traditional paper questionnaire to collect data. When the eligible caregivers took their children to community health service centres for regular health check-ups or vaccinations, health workers invited them to participate in the study. Health workers in the community health service centres first explained the survey’s aim to caregivers and obtained their written consent, after which the caregivers completed the questionnaire themselves. Staff from the Capital Institute of Pediatrics in Beijing performed as the supervisor. As soon as a caregiver completed the questionnaire, the supervisor immediately checked the completed questionnaire for any missing details or mistakes and made corrections with the caregiver before they left.

### Data management and analysis

We entered the paper questionnaires using EpiData, version 3.1, and checked them using the double-entry method. We conducted statistical analysis with SPSS, version 16.0, (IBM Corporation, Somers, New York, USA). We presented demographic characteristics as numbers and percentages.

### Reliability analysis

We used Cronbach's alpha coefficient and split-half reliability to assess the reliability of the scale [28]. Cronbach's alpha coefficient measured the internal consistency of the scale. It is regarded as reliable if the Cronbach’s alpha is >0.70 [29–31]. The split-half reliability was assessed by dividing the same scale item into two halves and calculating the simple correlation coefficient (r) for their scores, with a correlation coefficient >0.70 being considered acceptable [32].

### Validity analysis

#### Content validity

To ensure content validity, we constituted a multidisciplinary expert panel comprising eight scholars with specialisations in paediatrics (n = 1), child health care (n = 3), nutrition (n = 2), and psychology (n = 2), each possessing over a decade of clinical or research experience in their respective domains. All participating experts were asked to rate each item of the CCFPS on clarity (1 = not clear, 2 = item need some revision, 3 = clear but need minor revision, and 4 = very clear) and relevance (1 = not relevant, 2 = somewhat relevant, 3 = quite relevant, and 4 = highly relevant) [33]. We used content validity index (CVI) to assess the content validity, calculated by dividing the number of experts' ratings of 3 or 4 by the total number of experts. If CVI was >0.79, items were considered appropriate [34,35].

#### Structural validity

For structural validity, we carried out EFA with half of the survey sample (n = 464). Before the EFA, we checked the data set to ensure the data could be analysed under the ordinary conditions and adopted listwise deletion for missing data. We used the Kaiser-Meyer-Olkin value (KMO) and Bartlett's Test of Sphericity to evaluate whether the data fit factor analysis [36]. A KMO value of ≥0.5 and a *P*-value of <0.05 in the Bartlett test indicate appropriateness for factor analysis [36].

After ensuring the data's suitability, we employed parallel analysis to determine the optimal number of factors. This involved comparing the eigenvalues from the actual data with those from randomly generated data matrices. We also compared the results of parallel analysis with those of the competing model. The final factor model was determined by considering the model indices and the factor loadings after oblique rotation.

Subsequently, the factor structure constructed through EFA was the theoretical model and was tested with confirmatory factor analysis (CFA) [37]. We used the remaining half of the sample for the CFA [38]. We evaluated the adequacy of the model fit by four common fit indices: the χ^2^ statistic to degrees of freedom (χ2/df), comparative fit index (CFI), non-normed fit index (NNFI), and root mean square error of approximation (RMSEA). Good model fit is represented as χ2/df <3, NNFI>0.90, CFI>0.90, and RMSEA<0.08[39–42]. Finally, we applied principal component analysis to further validate each factor [43].

Furthermore, given that many factors influence the caregivers’ feeding practices, we also compared the scores in each dimension of CCFPS by regions, household structure, and socioeconomic status, including caregivers’ educational background, occupation, and family monthly income. We used means and standard deviations (SDs) to describe scores and considered two-tailed *P*-values of <0.05 for a significant difference.

## RESULTS

### Participant characteristics

A total of 928 caregivers with children aged 6–23 months completed the paper questionnaires between November 2016 and January 2017, of which 450 were aged 6–12 months and 478 were aged 12–23 months (Table S1 in the **Online Supplementary Document**). The ratio of male to female was close to 1:1. Nearly two-thirds of children were local residents (65.0%), and most primary caregivers were mothers (81.3%), followed by grandparents (16.6%), and others (2.2%). More than half of the primary caregivers held a college degree or above.

### Reliability

The internal reliability analysis included all 928 participants in the test sample. The Cronbach’s alpha coefficient for the total scale homogeneity reliability was 0.783, and 0.737–0.843 for dimensions (**Table 1**). The Guttman split-half reliability coefficient of the scale was 0.750, and 0.637–0.831 for dimensions.

**Table 1:** Reliability coefficients of the final CCFPS in all dimensions

	Cronbach’s alpha coefficient (n = 928)	Guttman Split-Half coefficient (n = 928)
**Pressure to eat**	0.759	0.637
**Concern about child undereating**	0.843	0.831
**Emotional feeding or instrumental feeding**	0.786	0.703
**Prompting and encouragement to eat**	0.737	0.699
**Restriction**	0.745	0.645
**Concern about child overeating**	0.804	0.795
**Total scale**	0.783	0.750

CCFPS – caregivers’ complementary feeding practice scale

### Validity

For content validity, the CVI for different dimensions ranged from 0.858 to 0.958 and the total CVI was 0.910, indicating appropriate content validity.

For structure validity, the result of the KMO test was 0.804, and Bartlett’s test of sphericity was statistically significant (χ^2^ = 3025.241; *P* < 0.001), meeting the factor analysis conditions. The parallel analysis plots and competing models were subjected to a comparative analysis, extracting six factors. All factor loadings were >0.4 (**Table 2**). Moreover, pressure to eat (R^2^ = 0.04586), concern about child undereating (R^2^ = 0.11758), emotional feeding or instrumental feeding (R^2^ = 0.07310), prompting and encouragement to eat (R^2^ = 0.17210), restriction (R^2^ = 0.05732), and concern about child overeating (R^2^ = 0.04837) collectively explained 51.433% of the variance in the 27 items.

**Table 2:** Factor loadings and variance contribution rate of the final CCFPS (n = 464)

Dimension (item numbers)	Factor loadings, min-max	Variance contribution rate, %
Pressure to eat (6, 7, 10)	0.411–0.737	4.586
Concern about child undereating (11, 12, 13, 23, 24, 26)	0.446–0.772	11.758
Emotional feeding or instrumental feeding (1, 2, 3, 21, 22)	0.552–0.761	7.310
Prompting and encouragement to eat (4, 5, 8, 9, 25, 27)	0.486–0.724	17.210
Restriction (17, 18, 19)	0.587–0.779	5.732
Concern about child overeating (14, 15, 16, 20)	0.464–0.780	4.837

CCFPS – caregivers’ complementary feeding practice scale

The results of CFA analysis showed χ^2^/df = 2.00, CFI = 0.915, NNFI = 0.904, and RMSEA = 0.046, indicating that the six-factor model demonstrated an acceptable fit (Table S2 in the **Online Supplementary Document**). Furthermore, we conducted principal component analyses on each factor, which revealed that there was only one eigenvalue >1 in each dimension, along with variance contribution rates ranging from 43% to 67% (**Table 3**). Loadings of all items were >0.5 within their dimensions.

**Table 3:** Results of principal component analysis for each dimension of CCFPS

Dimensions	Number of factors with eigenvalues >1	Variance contribution rate, %	Factor loadings, min-max
Pressure to eat	1	67.526	0.794–0.842
Concern about child undereating	1	54.772	0.608–0.782
Emotional feeding or instrumental feeding	1	54.385	0.622–0.826
Prompting and encouragement to eat	1	43.541	0.604–0.760
Restriction	1	66.369	0.795–0.852
Concern about child overeating	1	63.157	0.738–0.825

CCFPS – caregivers’ complementary feeding practice scale

There were no statistically significant differences in the scores across all dimensions of the CCFPS regarding household structure and occupation (Table S3 in the **Online Supplementary Document**). For regions, caregivers in Xiamen got a higher score for child undereating than those in Xi'an (*P* < 0.017), while no differences were observed in other dimensions. Regarding the average monthly family income, the group with a monthly income of CNY 10 000–20 000 was higher only in terms of their score of prompting and encouragement to eat than that of the group with a monthly income of CNY 2000–5000 (*P* < 0.001).

## DISCUSSION

This study conducted a rigorous scale evaluation and demonstrated that the CCFPS is a reliable and valid scale that can effectively assess caregivers’ complementary feeding practices of children aged 6–23 months in urban China.

For the reliability of the CCFPS, our results showed that the Cronbach’s alpha coefficient for the whole CCFPS and its dimensions were all >0.70, which indicated an acceptable level of internal consistency. The CCFPS scale has 27 items across six dimensions, each containing 3–6 items. These items play a crucial role in yielding the overall good alpha values. According to Nunnally, a sufficient number of items helps capture the construct comprehensively and increase the average inter-item correlation, thereby increasing the Cronbach's alpha coefficient [44]. Conversely, an insufficient number of items could lead to an incomplete representation of dimensions, resulting in a lower alpha value. The current item count appears optimal for achieving acceptable internal consistency, as seen in the scale's Cronbach's alpha coefficients (>0.70) for the whole scale and its dimensions. These findings suggest that the CCFPS scale was acceptable and reliable.

We employed content and structural validity to determine the validity of the CCFPS. By integrating expert opinions, it was determined that the CCFPS exhibited a high level of content validity (CVI = 0.910). In this study, the KMO measure was 0.84 and the Barlett Sphericity Test was significant (χ^2^ = 3025.241; *P* < 0.001), indicating the number of data points was suitable for CFA. Literature indicated that factor loadings should be >0.30 to consider the construct validity concerning sample size [45,46]. Our study found that items’ factor loadings ranged from 0.411 to 0.780, demonstrating structural validity. The scale’s factor structure was additionally evaluated using CFA, displaying a good database fit on the χ^2^/df = 2.00, CFI = 0.915, NNFI = 0.904, and RMSEA = 0.046, all meeting the appropriate requirements. Furthermore, the CCFPS in this study explained 51.433% of the total variance. It is reported that the variance explained in the scales should be ≥50% of the total variance, and the more variance is explained, the more accurately a concept or construct is assessed [47]. Therefore, these analyses above provided strong evidence supporting the strong structural validity of the CCFPS and demonstrated that the CCFPS was a legitimate instrument for assessing caregivers’ feeding practices in China.

Feeding practices of caregivers are critical in shaping the children’s eating behaviour. Responsive feeding is globally recommended and considered an important part of nurturing care [48], which has been proven to foster optimal growth and development and encourage children’s self-regulation, thus preventing both under- and overfeeding [49–52]. Conversely, non-responsive feeding, such as restricting food, pressure to eat, and using food as a reward, is associated children’s obesity [53–55]. Obesity among children and adolescents has become a global health concern [56]. The CCFPS evaluated in this study aligns with responsive feeding principles and could be used to assess whether caregivers adhere to guideline-based infant and young child feeding practices. By evaluating CCFPS, we can understand Chinese caregivers’ feeding practices and contribute to more inclusive and effective global efforts to combat childhood nutrition and obesity issues.

### Strengths and limitations

To our knowledge, the CCFPS is the first scale for assessing feeding practices of caregivers with children aged 6–23 months in China. However, there are some limitations to this study. First, the samples collected in this study exclusively comprised caregivers of infants and young children in urban areas, therefore, the scale may not be applicable to assess caregivers in rural areas. Second, due to the absence of an established 'gold standard' scale for comparison purposes, criterion validity testing could not be conducted. Future research could involve multi-centre studies. By aggregating data from diverse regions and populations, researchers can gradually establish a locally relevant and reliable criterion for validating the CCFPS. Moreover, subsequent studies should incorporate a convergent validity test by examining correlations with related constructs such as caregiver education, child nutritional status, or feeding frequency, to fully assess the construct validity of the scale.

## CONCLUSIONS

In this study, we used a standard methodology to assess the reliability and validity of the CCFPS. Our findings demonstrate that the CCFPS is a valid and reliable instrument, suitable for research or clinical practice in determining caregivers’ feeding practices with infants and young children aged 6–23 months in urban China.

## Additional Material


Online Supplementary Document

## References

[1] Likhar A Patil MS Importance of Maternal Nutrition in the First 1,000 Days of Life and Its Effects on Child Development: A Narrative Review. Cureus. 2022;14:e30083. 10.7759/cureus.30083 36381799 PMC9640361

[2] Pries AM Rehman AM Filteau S Sharma N Upadhyay A Ferguson EL Unhealthy Snack Food and Beverage Consumption Is Associated with Lower Dietary Adequacy and Length-for-Age z-Scores among 12-23-Month-Olds in Kathmandu Valley, Nepal. J Nutr. 2019;149:1843–51. 10.1093/jn/nxz140 31309223 PMC6768809

[3] Taylor CM Wernimont SM Northstone K Emmett PM Picky/fussy eating in children: Review of definitions, assessment, prevalence and dietary intakes. Appetite. 2015;95:349–59. 10.1016/j.appet.2015.07.026 26232139

[4] Wolstenholme H Kelly C Hennessy M Heary C Childhood fussy/picky eating behaviours: a systematic review and synthesis of qualitative studies. Int J Behav Nutr Phys Act. 2020;17:2. 10.1186/s12966-019-0899-x 31900163 PMC6942299

[5] Machado BC Dias P Lima VS Campos J Gonçalves S Prevalence and correlates of picky eating in preschool-aged children: A population-based study. Eat Behav. 2016;22:16–21. 10.1016/j.eatbeh.2016.03.035 27077700

[6] Shi C Li N Dong J Wang L Li X Ji C Association between maternal nonresponsive feeding practice and child’s eating behavior and weight status: children aged 1 to 6 years. Eur J Pediatr. 2017;176:1603–12. 10.1007/s00431-017-3007-8 28890989

[7] Wang S Huang X Wang H Jin X Prevalence of eating problems among 1-3 years children: A national survey in China. Chinese Journal of Child Health Care. 2012;20:109–11.

[8] Jin XM Shi R Jin ZJ [Epidemiological investigation on the eating problems of children aged 1 to 6 years in Shanghai]. Chinese Journal of Child Heal Care. 2009;17:387–9. Chinese.

[9] Lutter CK Grummer-Strawn L Rogers L Complementary feeding of infants and young children 6 to 23 months of age. Nutr Rev. 2021;79:825–46. 10.1093/nutrit/nuaa143 33684940

[10] Mahmood L Flores-Barrantes P Moreno LA Manios Y Gonzalez-Gil EM The Influence of Parental Dietary Behaviors and Practices on Children’s Eating Habits. Nutrients. 2021;13:1138. 10.3390/nu13041138 33808337 PMC8067332

[11] Pierron A Fond-Harmant L Laurent A Alla F Supporting parenting to address social inequalities in health: a synthesis of systematic reviews. BMC Public Health. 2018;18:1087. 10.1186/s12889-018-5915-6 30170577 PMC6119337

[12] Gray HL Buro AW Sinha S Associations Among Parents’ Eating Behaviors, Feeding Practices, and Children’s Eating Behaviors. Matern Child Health J. 2023;27:202–9. 10.1007/s10995-022-03572-6 36609937

[13] Fries LR Martin N van der Horst K Parent-child mealtime interactions associated with toddlers’ refusals of novel and familiar foods. Physiol Behav. 2017;176:93–100. 10.1016/j.physbeh.2017.03.001 28315360

[14] Holley CE Haycraft E Farrow C Unpacking the relationships between positive feeding practices and children’s eating behaviours: The moderating role of child temperament. Appetite. 2020;147:104548. 10.1016/j.appet.2019.104548 31809811

[15] Thompson AL Bentley ME The critical period of infant feeding for the development of early disparities in obesity. Soc Sci Med. 2013;97:288–96. 10.1016/j.socscimed.2012.12.007 23312304 PMC3812266

[16] Chen Q Zhang RZ Guo SY [The impact of family feeding patterns on obesity in adolescents and children]. Health Educ China. 2017;11:1024–1027. Chinese.

[17] World Health Organization. Consideration of the evidence on childhood obesity for the Commission on Ending Childhood Obesity: report of the ad hoc working group on science and evidence for ending childhood obesity, Geneva, Switzerland. Geneva, Switzerland: World Health Organization; 2016. Available: https://iris.who.int/bitstream/handle/10665/206549/9789241565332_eng.pdf. Accessed: 20 May 2025.

[18] NCD Risk Factor Collaboration (NCD-RisC) Worldwide trends in body-mass index, underweight, overweight, and obesity from 1975 to 2016: a pooled analysis of 2416 population-based measurement studies in 128·9 million children, adolescents, and adults. Lancet. 2017;390:2627–42. 10.1016/S0140-6736(17)32129-3 29029897 PMC5735219

[19] Birch LL Fisher JO Grimm-Thomas K Markey CN Sawyer R Johnson SL Confirmatory factor analysis of the Child Feeding Questionnaire: a measure of parental attitudes, beliefs and practices about child feeding and obesity proneness. Appetite. 2001;36:201–10. 10.1006/appe.2001.0398 11358344

[20] Hughes SO Power TG Orlet Fisher J Mueller S Nicklas TA Revisiting a neglected construct: parenting styles in a child-feeding context. Appetite. 2005;44:83–92. 10.1016/j.appet.2004.08.007 15604035

[21] Wardle J Sanderson S Guthrie CA Rapoport L Plomin R Parental feeding style and the inter-generational transmission of obesity risk. Obes Res. 2002;10:453–62. 10.1038/oby.2002.63 12055321

[22] Musher-Eizenman D Holub S Comprehensive Feeding Practices Questionnaire: validation of a new measure of parental feeding practices. J Pediatr Psychol. 2007;32:960–72. 10.1093/jpepsy/jsm037 17535817

[23] Baughcum AE Powers SW Johnson SB Chamberlin LA Deeks CM Jain A Maternal feeding practices and beliefs and their relationships to overweight in early childhood. J Dev Behav Pediatr. 2001;22:391–408. 10.1097/00004703-200112000-00007 11773804

[24] Thompson AL Mendez MA Borja JB Adair LS Zimmer CR Bentley ME Development and validation of the Infant Feeding Style Questionnaire. Appetite. 2009;53:210–21. 10.1016/j.appet.2009.06.010 19576254 PMC3130353

[25] Ma JQ Zhou L Hu Y Liu S Sheng X Association between feeding practices and weight status in young children. BMC Pediatr. 2015;15:97. 10.1186/s12887-015-0418-4 26306490 PMC4550067

[26] Lihui X. [Development and Evaluation of Parents’ Feeding Practice Scale for Infant and Young Child]. Beijing, China: Capital Institute of Pediatrics; 2017. Chinese.

[27] Tinsley HE Tinsley DJ Uses of factor analysis in counseling psychology research. J Couns Psychol. 1987;34:414–24. 10.1037/0022-0167.34.4.414

[28] Pronk T Molenaar D Wiers RW Murre J Methods to split cognitive task data for estimating split-half reliability: A comprehensive review and systematic assessment. Psychon Bull Rev. 2022;29:44–54. 10.3758/s13423-021-01948-3 34100223 PMC8858277

[29] Schermelleh-Engel K Moosbrugger H Müller H Evaluating the fit of structural equation models: Tests of significance and descriptive goodness-of-fit measures. Methods Psychol Res. 2003;8:23–74.

[30] Taber KS The use of Cronbach’s alpha when developing and reporting research instruments in science education. Res Sci Educ. 2018;48:1273–96. 10.1007/s11165-016-9602-2

[31] Ebadi A, Zarshenas L, Rakhshan M, Zareiyan A, Sharifnia S, Mojahedi M. Principles of scale development in health science. Tehran, Iran: Jame-e-negar; 2017.

[32] Xu J Shi Y Li S Ma J Zhang J Shen Y Development and reliability testing of a risk factor and risk outcome assessment scale for nurses in “internet + nursing services” for the elderly. BMC Nurs. 2024;23:54. 10.1186/s12912-024-01698-2 38238706 PMC10797920

[33] Zamanzadeh V Ghahramanian A Rassouli M Abbaszadeh A Alavi-Majd H Nikanfar AR Design and Implementation Content Validity Study: Development of an instrument for measuring Patient-Centered Communication. J Caring Sci. 2015;4:165–78. 10.15171/jcs.2015.017 26161370 PMC4484991

[34] Shrotryia VK Dhanda U Content Validity of Assessment Instrument for Employee Engagement. SAGE Open. 2019;9:2158244018821751. 10.1177/2158244018821751

[35] Melkamu Asaye M Gelaye KA Matebe YH Lindgren H Erlandsson K Assessment of content validity for a Neonatal Near miss Scale in the context of Ethiopia. Glob Health Action. 2021;14:1983121. 10.1080/16549716.2021.1983121 34694977 PMC8547862

[36] Polit DF, Beck CT. Nursing Research: Generating and Assessing Evidence for Nursing Practice. 10th ed. Philadelphia, Pennsylvania, USA: Wolters Kluwer; 2016.

[37] Tabachnick BG, Fidell LS, Ullman JB. Using multivariate statistics. 6th ed. Boston, Massachusetts, USA: Pearson; 2013.

[38] Tavakol M Wetzel A Factor Analysis: a means for theory and instrument development in support of construct validity. Int J Med Educ. 2020;11:245–7. 10.5116/ijme.5f96.0f4a 33170146 PMC7883798

[39] Albayrak S Çakır B Kılınç FN Vergili Ö Erdem Y Reliability and Validity Study of the Turkish Version of Child and Adolescent Social Support Scale for Healthy Behaviors. Asian Nurs Res (Korean Soc Nurs Sci). 2018;12:273–8. 10.1016/j.anr.2018.10.004 30394354

[40] Şimşek ÖF. Yapisal eşitlik modellemesine giriş - Temel ilkeler ve LISREL uygulamalari. Ankara, Turkey: Ekinoks; 2007.

[41] Lee KS Vaillancourt T Longitudinal Associations Among Bullying by Peers, Disordered Eating Behavior, and Symptoms of Depression During Adolescence. JAMA Psychiatry. 2018;75:605–12. 10.1001/jamapsychiatry.2018.0284 29641816 PMC6137525

[42] Palevych S Kirpenko V Piddubny A Bozhko S Tzymbaliyk Z Martinez Velez MA Structural validity of the physical fitness test battery. Health Sport Rehabilitation. 2021;7:84–97. 10.34142/HSR.2021.07.04.07

[43] Zhao TN Wang HW Liu GF [Confirmatory factor analysis and application]. Chin J Health Stat. 2010;27:608–9. Chinese.

[44] Nunnally JC. Psychometric theory. 2nd ed. New York, USA: McGraw-Hill; 1978.

[45] Yip MP Chang AM Chan J MacKenzie AE Development of the Telemedicine Satisfaction Questionnaire to evaluate patient satisfaction with telemedicine: a preliminary study. J Telemed Telecare. 2003;9:46–50. 10.1258/135763303321159693 12641893

[46] Martins BG Marôco J Barros MVG Campos J Lifestyle choices of Brazilian college students. PeerJ. 2020;8:e9830. 10.7717/peerj.9830 33083105 PMC7547619

[47] Yaşlıoğlu MM Factor analysis and validity in social sciences: Application of exploratory and confirmatory factor analyses. Istanbul University Journal of the School of Business. 2017;46:74–85.

[48] World Health Organization. WHO Guideline for complementary feeding of infants and young children 6-23 months of age. Geneva, Switzerland: World Health Organization; 2023. Available: https://iris.who.int/bitstream/handle/10665/373358/9789240081864-eng.pdf?sequence=1. Accessed: 20 May 2025.

[49] Health Eating Research. Feeding Guidelines for Infants and Young Toddlers: A Responsive Parenting Approach. Durham, North Carolina: Health Eating Research; 2017. Available: https://healthyeatingresearch.org/wp-content/uploads/2017/02/her_feeding_guidelines_report_021416-1.pdf. Accessed: 20 May 2025.

[50] Pérez-Escamilla R Segura-Pérez S Hall Moran V Dietary guidelines for children under 2 years of age in the context of nurturing care. Matern Child Nutr. 2019;15:e12855. 10.1111/mcn.12855 31240831 PMC7199077

[51] Sall NS Bégin F Dupuis JB Bourque J Menasria L Main B A measurement scale to assess responsive feeding among Cambodian young children. Matern Child Nutr. 2020;16:e12956. 10.1111/mcn.12956 31999399 PMC7296795

[52] Coleta H Schincaglia RM Gubert MB Pedroso J Factors associated with infant feeding styles in the Federal District, Brazil. Appetite. 2022;179:106290. 10.1016/j.appet.2022.106290 36058422

[53] Boucheron P Bhopal S Verma D Roy R Kumar D Divan G Observed feeding behaviours and effects on child weight and length at 12 months of age: Findings from the SPRING cluster-randomized controlled trial in rural India. PLoS One. 2020;15:e0237226. 10.1371/journal.pone.0237226 32790783 PMC7425856

[54] Beckers D Karssen LT Vink JM Burk WJ Larsen JK Food parenting practices and children’s weight outcomes: A systematic review of prospective studies. Appetite. 2021;158:105010. 10.1016/j.appet.2020.105010 33075443

[55] Bergamini M Simeone G Verga MC Doria M Cuomo B D’Antonio G Complementary Feeding Caregivers’ Practices and Growth, Risk of Overweight/Obesity, and Other Non-Communicable Diseases: A Systematic Review and Meta-Analysis. Nutrients. 2022;14:2646. 10.3390/nu14132646 35807827 PMC9268062

[56] Jebeile H Kelly AS O’Malley G Baur LA Obesity in children and adolescents: epidemiology, causes, assessment, and management. Lancet Diabetes Endocrinol. 2022;10:351–365. 10.1016/S2213-8587(22)00047-X 35248172 PMC9831747

