# Supplementary material for: Evaluation of the reliability and validity of a Caregivers’ Complementary Feeding Practice Scale (CCFPS) for children aged 6–23 months in urban areas of China
Source: J Glob Health. 2025 Jun 20;15:04170. doi: 10.7189/jogh.15.04170 (PMC12178594; doi:10.7189/jogh.15.04170)
Supplement: Online Supplementary Document [file jogh-15-04170-s001.pdf]

Supplement to: Wu Q, Meng N, Xie L, Li L, Huang Y, Zhang Y. Evaluation of the reliability and validity of caregivers' complementary feeding practice scale (CCFPS) for children aged 6–23 months in urban areas of China. J Glob Health. 2025;15:04170

Table S1. Characteristics of surveyed children and their primary caregivers

	n (%)
**Child's sex***	
Male	463 (49.9)
Female	462 (49.8)
**Child's age in months**	
6–11	450 (48.5)
12–24	478 (51.5)
**Household structure**	
Nuclear family	482 (51.9)
Lineal family	433 (46.7)
Other	13 (1.4)
**Primary caregiver**	
Mother	754 (81.3)
Grandparents	154 (16.6)
Other	20 (2.2)
**Caregivers' education**	
Primary school	33 (3.6)
Middle and high school	401 (43.2)
College	458 (49.4)
Postgraduate	36 (3.9)
**Caregivers' occupation**	
Household work	521 (56.1)
Other	407 (43.9)
**Family monthly income in CNY†**	
<2000	13 (1.4)

2000–5000	312 (35.4)
5000–10 000	349 (39.6)
10 000–20 000	158 (17.9)
>20 000	49 (5.6)
**Regions**	
Harbin	317 (34.1)
Xiamen	331 (35.7)
Xi'an	280 (30.2)

*Data missing for 3 participants.

†47 participants did not answer the income question.

Table S2. Selected goodness-of-fit indices from CFA models

Fit indices	Results	Tolerance interval
χ^2/df	2.00	<3
CFI	0.915	>0.9
NNFI	0.904	>0.9
RMSEA (90% CI)	0.046 (0.041–0.052)	<0.06

CFA – confirmatory factor analysis, CFI – comparative fit index, CI – confidence interval, df – degrees of freedom, NNFI – non-normed fit index, RMSEA – root mean square error of approximation, χ^2 – chi square

Table S3. Comparison of scores in each dimension of CCFPS among different regions, household structure, and income, x̄ (SD)

	Pressure to eat	Concern about child undereating	Emotional feeding or instrumental feeding	Prompting and encouragement to eat	Restriction	Concern about child overeating
**Regions**						
Harbin	2.74 (0.83)	2.52 (0.77)	2.85 (0.71)	3.74 (0.70)	3.64 (0.80)	2.65 (0.85)
Xiamen	2.84 (0.84)	2.76 (0.81)	2.79 (0.69)	3.72 (0.60)	3.59 (0.80)	2.43 (0.79)
Xi'an	2.76 (0.84)	2.59 (0.81)*	2.80 (0.72)	3.73 (0.65)	3.58 (0.83)	2.52 (0.83)
**Household structure**						

Nuclear family	2.73 (0.83)	2.58 (0.82)	2.80 (0.72)	3.72 (0.66)	3.65 (0.81)	2.50 (0.86)
Lineal family	2.80 (0.86)	2.61 (0.82)	2.82 (0.73)	3.74 (0.65)	3.52 (0.83)	2.56 (0.79)
**Education background**						
Primary school	2.67 (1.02)	2.89 (0.90)	3.03 (0.82)	3.41 (0.76)	3.23 (0.91)	2.17 (0.77)
Middle school	2.78 (0.90)	2.69 (0.82)	2.84 (0.67)	3.63 (0.62)	3.49 (0.85)	2.50 (0.82)
College	2.76 (0.79)	2.49 (0.80)	2.78 (0.74)	3.82 (0.66)†	3.67 (0.80)	2.57 (0.83)
Postgraduate	2.55 (0.77)	2.50 (0.71)	2.45 (0.77)	3.88 (0.57)	3.81 (0.73)	2.59 (0.88)
**Occupation**						
Household work	2.75 (0.85)	2.63 (0.79)	2.80 (0.72)	3.67 (0.65	3.56 (0.88)	2.50 (0.84)
Other	2.77 (0.83)	2.54 (0.84)	2.81 (0.73)	3.81 (0.65	3.61 (0.76)	2.56 (0.82)
**Family monthly income (in CYN)**						
<2000	2.67 (1.02)	2.47 (0.43)	2.92 (0.78)	3.69 (0.94)	2.97 (1.00)	2.46 (0.68)
2000–5000	2.70 (0.88)	2.67 (0.85)	2.79 (0.67)	3.61 (0.67)	3.50 (0.89)	2.49 (0.82)
5000–10 000	2.82 (0.85)	2.57 (0.80)	2.85 (0.77)	3.74 (0.64)	3.65 (0.77)	2.57 (0.86)
10 000–20 000	2.70 (0.79)	2.47 (0.78)	2.69 (0.72)	3.91 (0.57)‡	3.66 (0.80)	2.50 (0.86)
>20 000	2.86 (0.67)	2.54 (0.68)	2.83 (0.64)	3.88 (0.66)	3.65 (0.85)	2.57 (0.83)

CCFPS – caregivers' complementary feeding practice scale

*$P < 0.017$, compared with that of in Xi'an.

†$P < 0.003$, compared with that of middle school.

‡$P < 0.001$, compared with that of income 2000–5000.
